# ﻿Genus *Varma* (Hemiptera, Tropiduchidae): new species description and updated male and female identification keys

**DOI:** 10.3897/zookeys.1245.150663

**Published:** 2025-07-15

**Authors:** Huan Zhou, Li He, Xiang-Sheng Chen, Zhi-Min Chang

**Affiliations:** 1 Institute of Entomology, College of Agriculture, Guizhou University, Guiyang, Guizhou, 550025, China Guizhou University Guiyang China; 2 The Provincial Special Key Laboratory for Development and Utilization of Insect Resources, Guizhou University, Guiyang, Guizhou, 550025, China Guizhou University Guiyang China; 3 The Provincial Key Laboratory for Agricultural Pest Management of Mountainous Regions, Guizhou University, Guiyang 550025, China Guizhou University Guiyang China

**Keywords:** Identification key, morphology, new species, planthopper, taxonomy, typical tropiduchids, *Varma*

## Abstract

A new species of *Varma* Distant, 1906, *Varmamicroprojecta* Zhou & Chang, **sp. nov.** (China: Yunnan) is described and illustrated. The diagnosis of the genus is updated. A checklist and updated male and female identification keys to the species of *Varma* in China are provided.

## ﻿Introduction

The planthopper family Tropiduchidae Stål, 1866 is a relatively small group within the superfamily Fulgoroidea (Hemiptera, Fulgoromorpha), comprising 683 species in 198 genera (Bourgoin 2025). The family is divided into two subfamilies: Tropiduchinae Stål, 1866 and Elicinae Melichar, 1915, based on the shapes of gonapophyses IX and gonoplacs in female genitalia (Gnezdilov 2013). The Tropiduchinae, so-called “typical tropiduchids”, with a triangular posterior connective lamina of gonapophyses IX and an elongate gonoplacs, represents the larger group with 522 species in 147 genera distributed across 19 tribes (Gnezdilov 2013; Bourgoin 2025). The tribe Tropiduchini Stål, 1866 is the largest in Tropiduchinae, comprising 119 species in 27 genera. Morphological, phylogenetic, and zoogeographic analyses of this tribe by Wang et al. (2016) reveal that Tropiduchini represents a monophyletic group, supported by morphological synapomorphies including an asymmetrical pygofer and gonostyli. The *Varma*+ clade is identified as the probable oldest lineage within the tribe, with a likely origin in continental China.

The genus *Varma* Distant, 1906 was established with the type species *Seridafervens* Walker, 1857 from Borneo (Distant 1906). To date, nine species of this genus are known (Bourgoin 2025), of which five species have been reported in China, including *V.gibbosa* Wang & Liang, 2008, *V.bimaculata* Wang & Liang, 2008, *V.serrata* Men & Qin, 2010, *V.falcata* Chang & Chen, 2014 and *V.lobata* Chang & Chen, 2014 (Wang and Liang 2008; Men et al. 2010; Chang et al. 2014). Little attention had been given to female genitalia in species identification until Chang et al. (2014) demonstrated the diagnostic value of the endogonocoxal lobe and sternite VII.

This paper aims to describe a new species, *Varmamicroprojecta* Zhou & Chang, sp. nov., from Yunnan, China, and to provide updated identification keys to Chinese *Varma* species for both males and females, based on the first collection of female specimens of *Varmabimaculata* Wang & Liang, 2008 and including the newly described species.

## ﻿Material and methods

The external morphology was observed under a stereo microscope. The insect’s body size was measured using a Nikon SMZ25 digital imaging system. All measurements are in millimeters (mm). Habitus images were taken using a Canon 5D Mark IV digital camera with an MP-E 65 mm f/2.8 1–5× macro lens and a Godox MF12 flash as the light source. Multiple layers were stacked using Zerene Stacker v. 1.04. Abdomens of the examined specimens were removed and macerated in 10% KOH overnight, washed in water, and then transferred into glycerine. Genitalia were observed and illustrated under a Leica MZ 12.5 stereomicroscope. The photographs and illustrations were imported into Adobe Photoshop v. 23.0.0 for labeling and plate composition.

The external morphological terminology follows Bourgoin and Huang (1990) and Bourgoin et al. (2015), and the terminology for female genitalia follows Bourgoin (1993) and Song et al. (2024) for male genitalia. The type specimens are deposited in the Institute of Entomology, Guizhou University, Guiyang, China (GUGC). The metatibiotarsal formula lT-(aT-d)/aI/aII corresponds to the number of lateral teeth (lT) and apical teeth (aT) on the metatibia bearing a diastema (d), the number of apical teeth on the first (aI) and second (aII) metatarsomere.

## ﻿Taxonomy

### ﻿Family Tropiduchidae Stål, 1866

**Subfamily Tropiduchinae Stål, 1866**

**Tribe Tropiduchini Stål, 1866**

#### ﻿Genus Varma

Taxon classificationAnimaliaHemipteraTropiduchidae

Distant, 1906

5410A0D5-0835-5663-B8A7-413C3AACDC49

Varma Distant, 1906: 330; Distant 1909: 171; Melichar 1914: 117; Wang and Liang 2008: 116; Men et al. 2010: 93; Chang et al. 2014: 23.

##### Type species.

*Varmafervens* Walker, 1857, Distant 1906: 330, by original designation.

##### Diagnosis.

See Chang et al. (2014), with in addition, the spinulation of the hind leg is 3-(6(5)-0)/5(6)/2. The gonapophyses VIII (first valvular) of the female genitalia has 2–4 distinct teeth on the ventral margin (e.g., Fig. 2D, 4D).

##### Distribution.

Oriental region, including South China, India, Indonesia, Malaysia, Sri Lanka (Fig. 5).

### ﻿Checklist of *Varma* Distant, 1906 in China

*V.bimaculata* Wang & Liang, 2008; Xizang

*V.falcata* Chang & Chen, 2014; Guizhou

*V.gibbosa* Wang & Liang, 2008; Xizang

*V.lobata* Chang & Chen, 2014; Yunnan

*V.microprojecta* Zhou & Chan, sp. nov.; Yunnan

*V.serrata* Men & Qin, 2010; Hunan, Yunnan

### ﻿Key to the species of genus *Varma* Distant, 1906 in China (modified from Chang et al. 2014)

#### ﻿Based on male genitalia, ♂

**Table:** 

1	Pygofer with posterior margin produced into a distinct process on the right side	**2**
–	Pygofer without such a process	**4**
2	Right posterior margin process of pygofer trapezoidal	**3**
–	Right posterior margin process of pygofer produced into a strip (see Chang et al. 2014: figs 29, 32)	***V.lobata* Chang & Chen**
3	Aedeagus with a pediform flat plate and wing-shaped lobe on the right side (see Men et al. 2010: fig. 2G)	***V.serrata* Men & Qin**
–	Aedeagus with a fusion of two semicircular plate-like structures and a glove-like structure on the right side (Fig. 3J, K)	***V.microprojecta* Zhou & Chang, sp. nov.**
4	Gonostyli with a subcircular or subglobose lobe at apical inner margin	**5**
–	Gonostyli with a falcate lobe at apical inner margin (see Chang et al. 2014: fig. 11)	***V.falcata* Chang & Chen**
5	Aedeagus with apical part expanded into two hemispherical protuberances, curved through about 180 degrees (see Wang and Liang 2008: figs 8, 9)	***V.gibbosa* Wang & Liang**
–	Aedeagus with apical part expanded into a hemispherical protuberance, curved through about 90 degrees, then extended into an irregularly contorted scoop-shape plate (see Wang and Liang 2008: figs 21, 22)	***V.bimaculata* Wang & Liang**

#### ﻿Based on female genitalia, ♀

**Table:** 

1	Posterior margin of sternite VII with a median triangular projection	**2**
–	Posterior margin of sternite VII relatively straight or with a median pit	**3**
2	Sternite VII with a median, posteriorly directed symmetrical triangular projection; endogonocoxal lobe with paw-like protrusions, broader on left side, slender on right (see Chang et al. 2014: fig. 17)	***V.falcata* Chang & Chen**
–	Sternite VII with asymmetrical median triangular projection directed left posterior; endogonocoxal lobe with left finger-like and right beak-like protrusions (Fig. 2C)	***V.bimaculata* Wang & Liang**
3	Sternite VII without median pit; endogonocoxal lobe extending irregularly in a triangular protrusion (see Chang et al. 2014: fig. 36)	***V.lobata* Chang & Chen**
–	Sternite VII with a median pit	**4**
4	Sternite VII with a semicircular shallow pit in middle, endogonocoxal lobe extending irregularly in a left triangular and right falculate protrusions (see Chang et al. 2014: fig. 20)	***V.gibbosa* Wang & Liang**
–	Sternite VII with a barrel-shaped pit in middle, endogonocoxal lobe extending asymmetrically in irregular stripes	**5**
5	Endogonocoxal lobe produced in irregular stripes with triangular projection on the right side (see Chang et al. 2014: fig. 39)	***V.serrata* Men & Qin**
–	Endogonocoxal lobe produced irregular stripes without triangular projection on the right side (Fig. 4C)	***V.microprojecta* Zhou & Chang, sp. nov.**

##### ﻿ Varma bimaculata

Taxon classificationAnimaliaHemipteraTropiduchidae

Wang & Liang, 2008

58F45D7D-B34B-51F6-978A-760688E9B437

Figs 1A–B , 2A–H

Varma bimaculata Wang & Liang, 2008: 120.

###### Material examined.

• 2♂♂, 3♀♀, **China** Xizang, Beibeng (29°14'26"N, 95°10'29"E), 13 Aug. 2022, Y.-J. Sui.

###### Distribution.

China (Xizang).

###### Description.

See Wang and Liang 2008

***Female genitalia*.** Genitalia symmetrical on both sides, except for endogonocoxal lobe and sternite VII (Fig. 2A, C). Anal tube (Fig. 2A) symmetrical elongate, with anal styles not exceeding its apex. Gonapophyses VIII (first valvular) (Fig. 2B, D) strongly sclerotized, saw-like, with 7 distinct teeth on the dorsal margin, 2 distinct teeth on the ventral margin, numerous small, indistinct teeth along both margins. Gonapophyses IX (second valvula) (Fig. 2E–G) reduced, triangular, bilaterally symmetrical, lateral margins somewhat sclerotized, remainder largely membranous. Gonoplace (third valvula) (Fig. 2B, H) broad, membranous, with about 12 teeth on the ventral margin and apical margin. Endogonocoxal lobe (Fig. 2C) at the base of the gonapophyses VIII produced mesad finger-like on the left side and beak-like on the right side. Posterior margin of sternite VII (Fig. 2C) with asymmetrically median triangular projection, the tip of process directed to the left posterior in ventral view.

**Figure 1.**
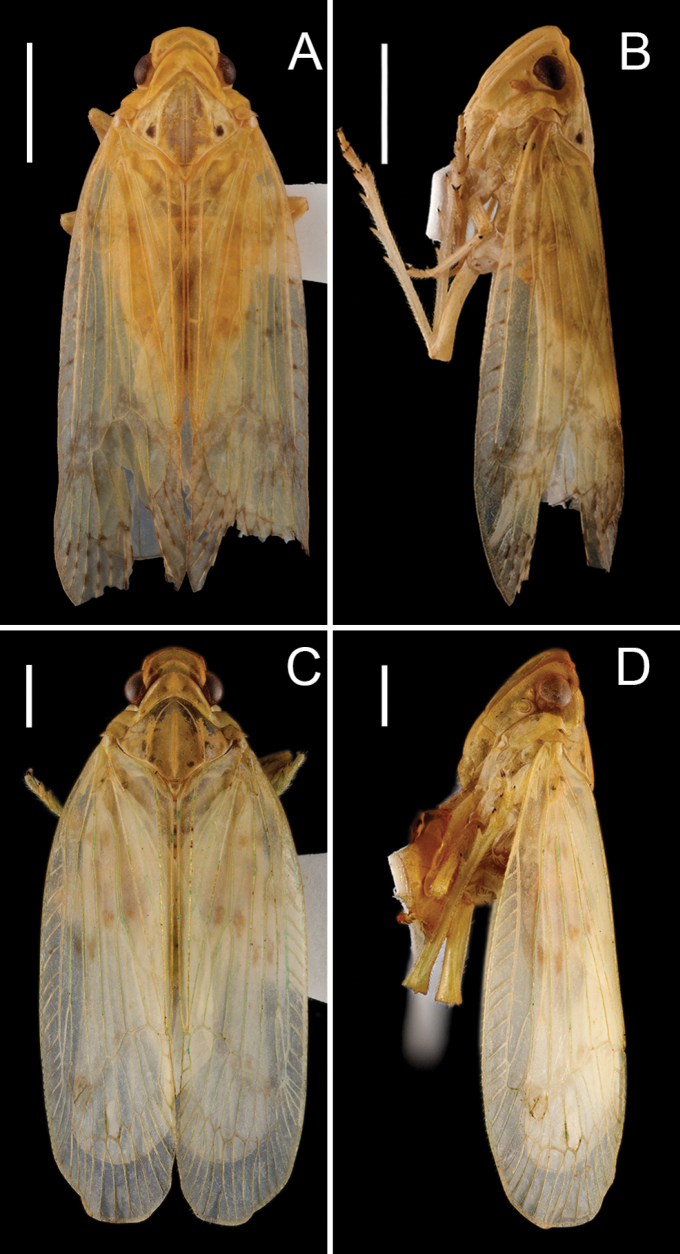
Male habitus of *Varma* species. **A, B.***Varmabimaculata* Wang & Liang, 2008; **C, D.***Varmamicroprojecta* Zhou & Chang, sp. nov.; **A, C.** Dorsal view; **B, D.** Lateral view. Scale bars: 1 mm.

**Figure 2.**
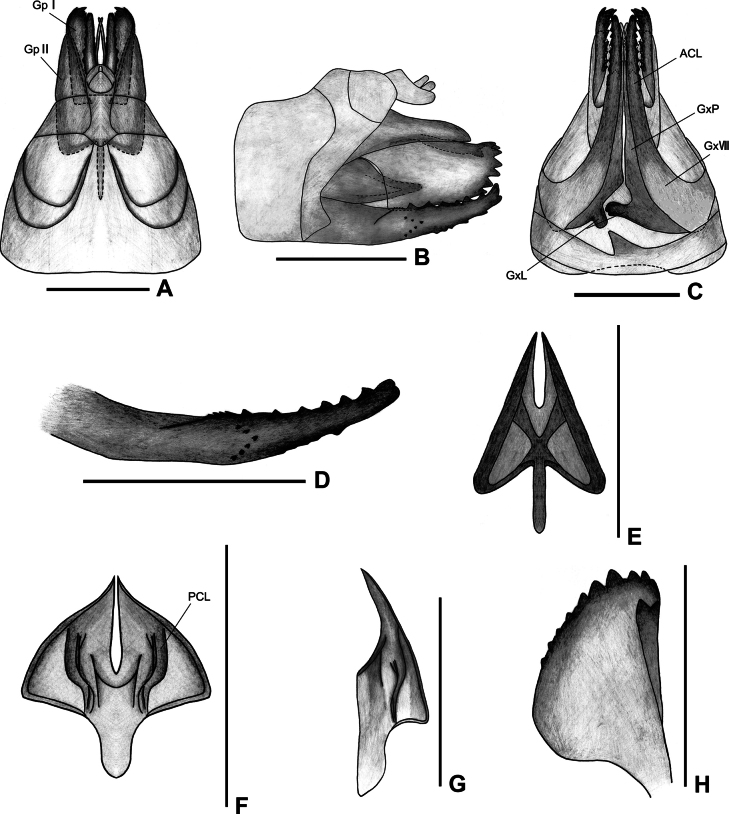
Female genitalia of *Varmabimaculata* Wang & Liang. **A.** Dorsal view; **B.** Lateral view; **C.** Ventral view; **D.** Gonapophyses VIII, lateral view; **E.** Gonapophyses IX, ventral view; **F.** Gonapophyses IX, dorsal view; **G.** Gonapophyses IX, lateral view; **H.** Gonoplace, inner view from the apex. Abbreviations: ACL, anterior connective lamina of gonapophyses VIII; Gp I, first lobe (lateral lobe) of gonoplace; Gp II, second lobe (posterior lobe) of gonoplace; GxL, endogonocoxal lobe; GxP, endogonocoxal process; Gx VIII, gonocoxae VIII; PCL, posterior connective lamina. Scale bars: 1 mm.

###### Remarks.

The female genitalia of this species are similar to those of *V.falcata*, but can be distinguished by the posterior margin of sternite VII bearing an asymmetrically triangular projection pointing to the left posterior in ventral view (vs a symmetrical median triangular projection, directed to middle posterior in *V.falcata*). Additionally, the endogonocoxal lobe has a finger-like protrusion on the left side and a beak-like protrusion on the right side (vs broader paw-like protrusions on the left and slender ones on right side in *V.falcata*).

##### ﻿ Varma microprojecta

Taxon classificationAnimaliaHemipteraTropiduchidae

Zhou & Chang sp. nov.

03CD79E9-8C23-5136-A976-9E4213E283BB

https://zoobank.org/745D3A19-7A5C-4B9B-8B75-F29463CB0441

**Figure 3.**
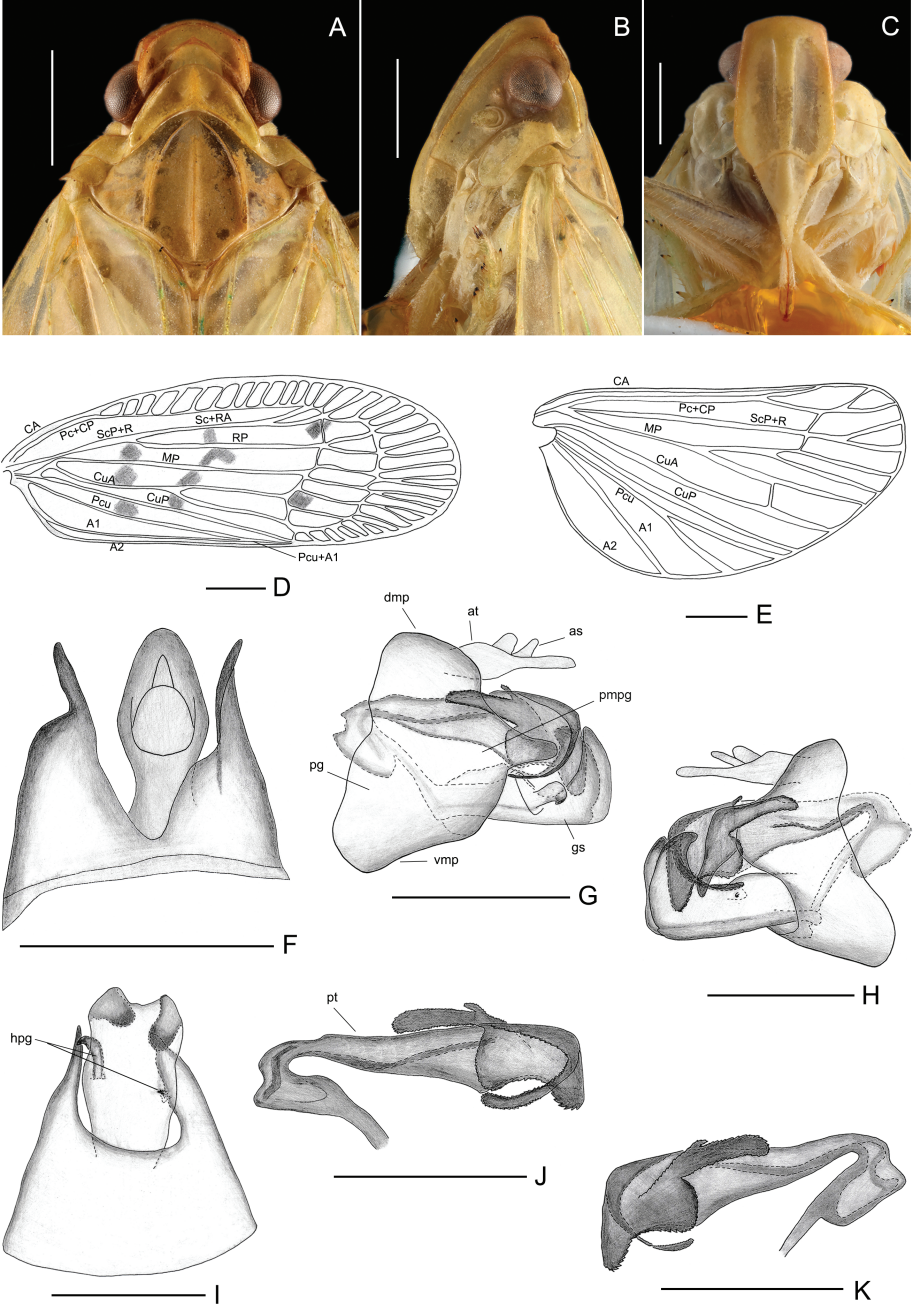
*Varmamicroprojecta* Zhou & Chang, sp. nov. male. **A.** Head and thorax, dorsal view; **B.** Head and thorax, left view; **C.** Head and thorax, ventral view; **D.** Forewing; **E.** Hindwing; **F.** Pygofer and anal tube, dorsal view; **G.** Male genitalia, left view; **H.** Male genitalia, right view; **I.** Pygofer and genital styles, ventral view; **J.** Aedeagus, left view; **K.** Aedeagus, right view. Abbreviations: as, anal style; at, anal tube (segment X); dmp, dorsal margin of pygofer; gs, gonostyli; hpg, hook-like process of gonostyli; pg, pygofer; pmpg, posterior margin of pygofer; pt, phallotheca; vmp, ventral margin of pygofer. Scale bars: 1 mm.

**Figure 4.**
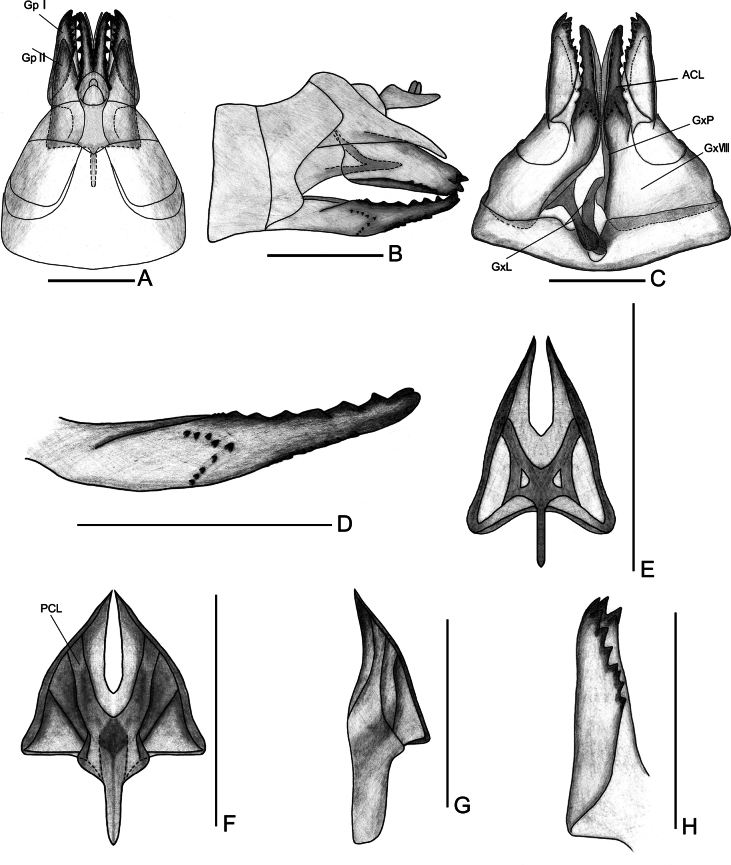
Female genitalia of *Varmamicroprojecta* Zhou & Chang, sp. nov. **A.** Dorsal view; **B.** Lateral view; **C.** Ventral view; **D.** Gonapophyses VIII, lateral view; **E.** Gonapophyses IX, ventral view; **F.** Gonapophyses IX, dorsal view; **G.** Gonapophyses IX, lateral view; **H.** Gonoplace, inner view from the apex. Abbreviations: ACL, anterior connective lamina of gonapophyses VIII; Gp I, first lobe (lateral lobe) of gonoplace; Gp II, second lobe (posterior lobe) of gonoplace; GxL, endogonocoxal lobe; GxP, endogonocoxal process; Gx VIII, gonocoxae VIII; PCL, posterior connective lamina. Scale bars: 1 mm.

Figs 1C–D , 3A–K , 4A–H

###### Type material.

***Holotype***: • ♂, **China** Yunnan Province, Baoshan City, Baihualing National Nature Reserve (25°17'22"N, 98°48'25"E), 6 Aug. 2013, W.-C. Yang. ***Paratypes***: • ♂, same data as holotype, 5 Aug. 2013, W.-C. Yang; • ♂, same data as holotype, 4 Aug. 2013, Z.-H. Fan; • 2♀♀, same data as holotype, 6 Aug. 2013, W.-C. Yang and H-Y. Sun.

###### Description.

***Measurements*.** Body length (from apex of vertex to tip of forewings): male 8.0–8.8 mm (*N* = 3), female 9.4–9.5 mm (*N* = 2).

***Coloration*.** General color pale green to stramineous yellow. Vertex, pronotum and mesonotum grayish green to pale ocherous. Forewings pale green or pale yellow, with nine irregular brownish spots around basal and middle part, and near nodal lines. Hind wings transparent.

***Head and thorax*.** Vertex (Figs 1C, 3A) unicarinate, distinctly broader than long in middle line (2.9: 1), projecting in front of the eyes; disc depressed; anterior margin arched and convex; posterior margin triangularly concave. Frons (Fig. 3C) with stout median carina, distinctly longer medially than its maximum width. (1.4: 1), lateral margins subparallel, with an arched frontoclypeal sulcus. Clypeus triangular, broadly rounded, with stout median carina, without lateral carinae (Fig. 3C). Pronotum (Figs 1C, 3A) tricarinate, wider than long in middle (4.0: 1) and longer than vertex in middle (1.4: 1), anterior margin distinctly arched convexly, posterior margin obtusely concave. Mesonotum (Figs 1C, 3A) tricarinated, wider than long in middle (1.4: 1), and longer than vertex and pronotum together (1.6: 1), median carina straight, reaching to mesoscutellum, lateral carinae curving anteriorly towards median carina. Forewing (Fig. 3D) subhyaline, 2.6 times longer than widest breadth (2.6: 1), anterior and posterior margins parallel, costal cell with 13 oblique transverse veins, ScP+R vein forked near basal 1/4, MP vein simple, CuA forked two branches in basal 1/3, CuP simple, claval veins Pcu and A_1_ uniting in middle of tegmina, nodal line and subapical line distinct. Hind wing hyaline, venation simple as in Fig. 3E. The spinulation of the hind leg is 3-(6-0)/6/2.

***Male genitalia*.** Anal tube symmetrical, long, its base narrow, widest in middle; anal style relatively small, not exceeding the tip of the anal tube (Fig. 3F–H). Pygofer (Fig. 3F–I) asymmetrical, left side (Fig. 3F, G) irregularly broad and triangular, with the posterior margin produced caudally into short finger-like processes near the middle; right side subquadrate, with the posterior margin bearing quadrilateral processes. Gonostyli asymmetrical, falciform in lateral view, bearing a large hook-like process on the left side, and a smaller one on the right side (Fig. 3G–I). In ventral view, gonostyli basal 2/3 fused, with micro projection on the apical margin (Fig. 3I). Aedeagus (Fig. 3G, H, J, K) relatively long, tubular, narrow at base and expanded apex, divided into two parts at the apex, one part ribbon-like with jagged edges, the other part with fusion of two semicircular projection and a glove-like projection, both with serrated margins. Phallotreme fissure-like, exposed in middle. Corpus connectivi robust, tubular (Fig. 3G, H, J, K).

***Female genitalia*.** Genitalia bilaterally symmetrical, except for endogonocoxal lobe and sternite VII (Fig. 4A, C). Anal tube symmetrical, long, base narrow, widest in middle, style small, not exceed the apex of anal tube (Fig. 4A, B). Gonapophyses VIII (first valvula) (Fig. 4B, D) saw-like, strongly sclerotized, with 7 distinct teeth on the dorsal margin, 4 distinct teeth on the ventral margin, and numerous indistinct, small teeth along both margins. Gonapophyses IX (second valvula) (Fig. 4E–G) reduced, triangular, bilaterally symmetrical, lateral margins somewhat sclerotized, remainder largely membranous. Gonoplace (third valvula) (Fig. 4B, H) broad, membranous, with 11 teeth on the ventral margin and apical margin. Endogonocoxal lobe (Fig. 4C) at the base of gonapophyses VIII produced mesad irregular stripes, left stripe straight, with apical part somewhat extension, and right stripe cambered, apical part swollen. Posterior margin of sternite VII (Fig. 4C) with an asymmetrical median deep pit, forming two irregular protrusions in ventral view.

###### Host plant.

Unknown.

###### Etymology.

The name of the new species is given for the presence of a microprojection on the apical margin of the gonostyli.

###### Distribution.

China (Yunnan).

###### Remarks.

This new species resembles *V.serrata* Men & Qin, 2010, but is distinguished from the latter by: 1) posterior margin of pygofer with quadrilateral processes on the right side (vs posterior margin of pygofer with trapezoidal lobe on the right side in *V.serrata*); 2) aedeagus of male genitalia with a slender ribbon-like projection on the left side, one part with a fusion of two semicircular plate-like projection on the upper part and a glove-like projection on the right side, both with serrated margins (vs absent in *V.serrata*); 3) gonostyli with a micro projection on the apical margin (but with a semicircular lobe in *V.serrata*); and 4) endogonocoxal lobe of female genitalia with smooth stripe on the right side (vs with a triangular projection in *V.serrata*).

## ﻿Discussion

The new species described in this paper shares key diagnostic characteristics of the genus, including a vertex wider than long, with a distinct median carina not reaching the anterior margin, a broad median carina on the frons, bifurcation of the ScP+R and CuA veins, the MP vein reaching the nodal, and the presence of both distal cells and subdistal cells. These features support its placement within the genus. The genitalia of this genus *Varma* (including the new species described here) are largely similar, with variations primarily occurring in: 1) the apical inner lobe of the gonostyli and the aedeagal projection for the male, and 2) the morphology of the posterior margin of the sternite VII and endogonocoxal lobe for the female. Additionally, for the new species described here, male and female genitalia resemble those of *V.serrata*. This study also provides a supplementary description of the female genitalia of *V.bimaculata*, whose endogonocoxal lobe and the structure of sternite VII appear stable and different from those of other species in the genus, further supporting the diagnostic value of female genital structures for species-level identification.

**Figure 5.**
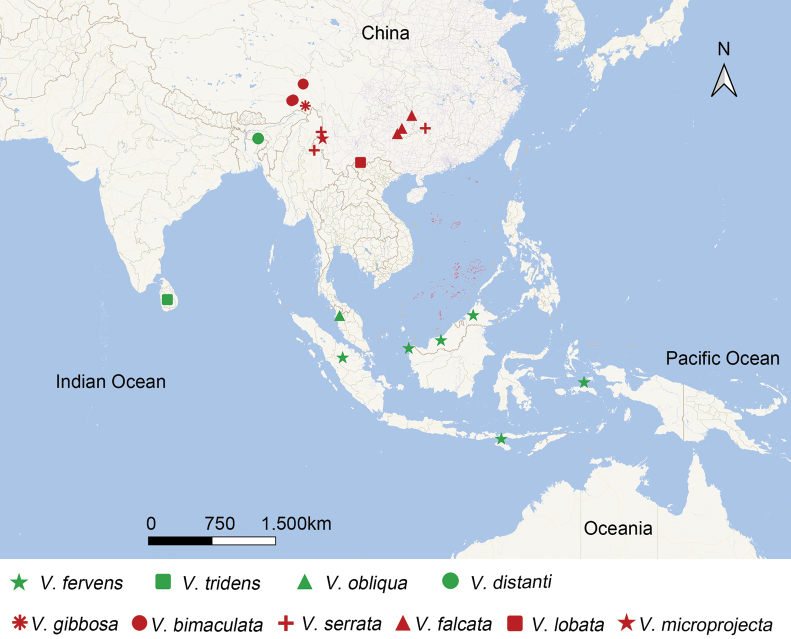
Geographical distribution of *Varma* species. Red icons indicate distribution in China, green icons indicate distribution in other countries.

The genus *Varma* is relatively small, with only ten species, of which six species are distributed in China, including the new species described in this paper. The remaining four species were distributed in Borneo, Sri Lanka, the Malaysian Peninsula, and India (Fig. 5). Wang et al. (2016) previously suggested that the genus may have originated from continental China. Indeed, the majority of known species of this genus are distributed in this region, and a distribution map compiling current records is presented in Fig. 5.

This map also shows that the non-Chinese species are geographically proximate to continental China, which may support the hypothesis of a Chinese origin followed by dispersal through stepping-stone colonization or tectonic movements (Wang et al. 2016). However, this hypothesis remains to be tested, and currently lacks robust supporting evidence. Further research, including expanded distributional surveys and molecular analyses that confirm the morphological phylogenetic framework of Wang et al. (2016) are needed to test this biogeographic scenario.

## Supplementary Material

XML Treatment for Varma

XML Treatment for Varma bimaculata

XML Treatment for Varma microprojecta

## ﻿Additional information

### Use of AI

No use of AI was reported.

### Author ORCIDs

Huan Zhou https://orcid.org/0009-0000-6887-8221

Li He https://orcid.org/0009-0003-3079-3013

Xiang-Sheng Chen https://orcid.org/0000-0001-9801-0343

Zhi-Min Chang https://orcid.org/0000-0002-7247-9706

### Data availability

All of the data that support the findings of this study are available in the main text.
